# Activation of 5-HT1A autoreceptors causes actin depolymerization in *Drosophila* serotonergic neurons.

**DOI:** 10.17912/micropub.biology.002024

**Published:** 2026-03-01

**Authors:** Ava Kinser, Jessie K. Rhodes, Douglas H. Roossien

**Affiliations:** 1 Biology, Ball State University, Muncie, IN, US; 2 Biological Sciences, Mount Holyoke College, South Hadley, MA, US; 3 Biological Sciences; Neuroscience and Behavior, Mount Holyoke College, South Hadley, MA, US

## Abstract

Serotonergic neurons extend long, highly branched axons throughout the brain during development and are responsible for the modulation of many behaviors. Because proper behavioral output is dependent on precise outgrowth and targeting of serotonergic axons, it is important to understand how serotonergic axon outgrowth is regulated during development. Our previous pharmacological experiments suggest that autoreceptor 5-HT1A negatively regulates axon outgrowth and branching of *Drosophila* serotonergic neurons *in vitro* , though the cellular mechanisms are unknown. Here we show that pharmacological activation of 5-HT1A leads to increases in G/F-actin ratios, suggesting 5-HT1A negatively regulates serotonergic axon outgrowth through actin depolymerization.

**Figure 1. Serotonin treatment and pharmacological activation of serotonin receptor 5-HT1A increase G/F-actin ratios in serotonergic neurons:**
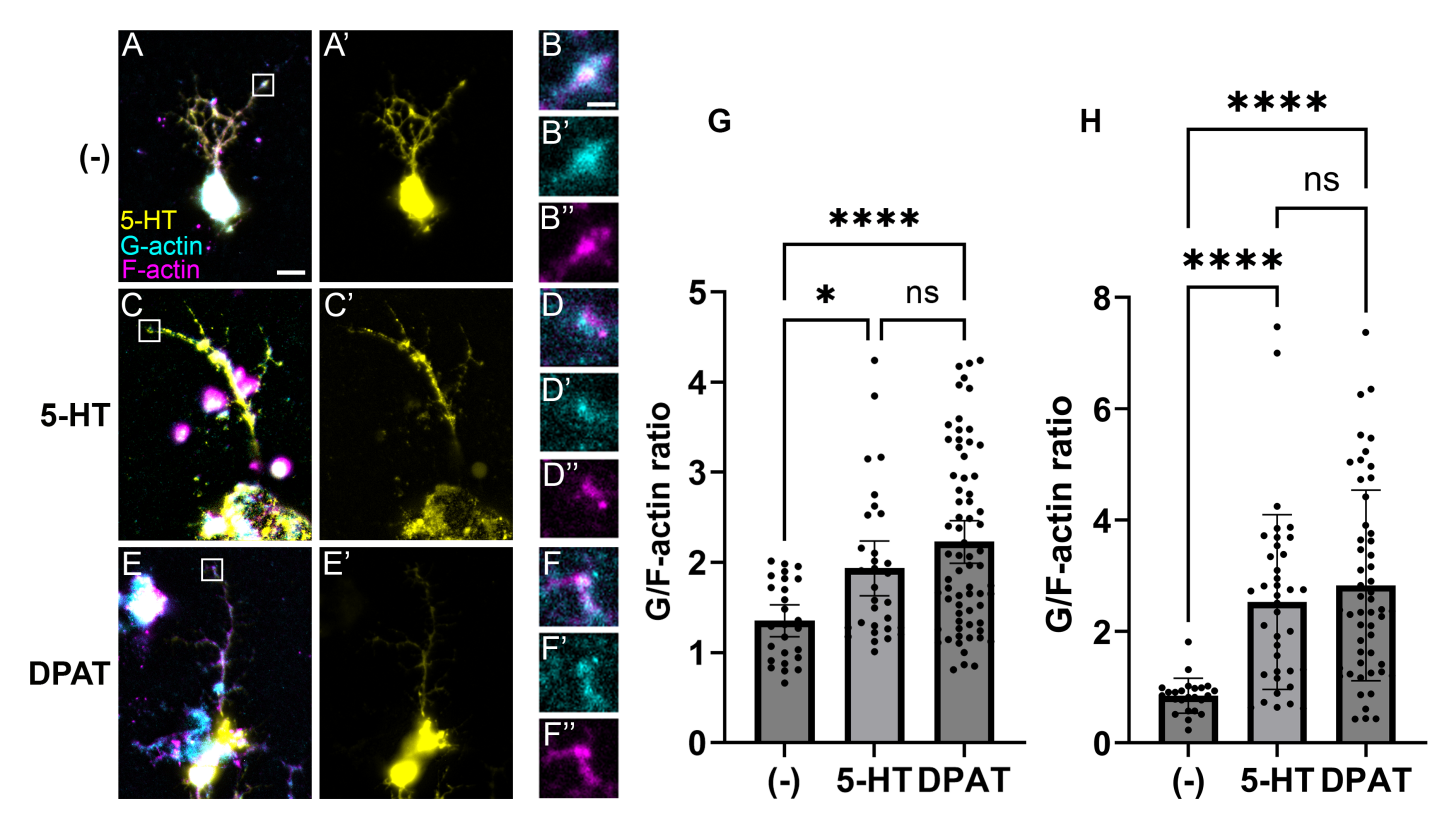
Neurons were stained using α5-hydroxytryptamine (5-HT) antibodies, SiR-Actin (F-actin), and DNAseI (G-actin). ( **A** , **C** , **E** ) Fluorescent images of serotonergic neurons with 5-HT, G-actin, and F-actin channels merged. (a’,c’,e’) show serotonergic neurons identified by positive α5-HT signal. ( **B** , **D** , **F** ) Corresponding distal axon regions (boxes in A, C, E). ( **G** ) G/F-actin ratios calculated from epifluorescent images. ( **H** ) G/F-actin ratios calculated from maximum projections of Z-stacks. * *p* -Value <.05, ** *p* -value <.01, *** *p* -value <.005 by Kruskal–Wallis&nbsp; *H* test. Scale bar in A = 10&nbsp;μm. Scale bar in B = 2.5&nbsp;μm.

## Description

In addition to its canonical role as a neurotransmitter, serotonin (5-hydroxytryptamine or 5-HT) negatively autoregulates the outgrowth of serotonergic axons (Budnik et al., 1989; Daubert & Condron, 2010; Diefenbach et al., 1995; Migliarini et al., 2013). Previous experiments in primary *Drosophila* serotonergic neurons showed this occurs through 5-HT1A receptor signaling (Long et al., 2023). Growth cones at the distal tip of actively growing axons contain radially arranged bundles of F-actin, which aid in adhesion, navigation, and elongation of the axon (Miller & Suter, 2018). In non-serotonergic snail growth cones, addition of 5-HT causes F-actin depolymerization (Torreano et al., 2005). We therefore hypothesized that activation of 5-HT1A negatively regulates axon outgrowth in serotonergic neurons by inducing actin depolymerization. Here we tested this hypothesis using a previously established fluorescence method of estimating the ratios of G-actin and F-actin (Torreano et al., 2005) after pharmacological treatment with 5-HT or the 5-HT1A agonist 8-OH-DPAT (DPAT).

Primary serotonergic neurons were harvested from Stage 11 *Drosophila* embryos, the point at which serotonergic neurons are committed to their cell fate and are undergoing active periods of axon outgrowth (Chen & Condron, 2008; Vallés & White, 1988). Neurons were cultured for 24 hours then treated 2 hours with either negative control media, 5-HT, or DPAT. The neurons were then stained with α5-HT, SiR-Actin (F-actin stain), and DNAseI (G-actin stain) to identify serotonergic neurons and estimate F- and G- actin levels by creating ROIs as the most distal 5 μm ends of axons in each channel. The samples were imaged on a Zeiss Apotome, which is capable of both standard epifluorescent imaging and optical sectioning. Using standard epifluorescence images, we found the average G/F actin ratio in controls was 1.35 (n = 25 ROIs measured from 11 neurons), whereas treatment with 5-HT and DPAT increased the average ratios to 2.01 (n = 31 ROIs measured from 22 neurons) and 2.23 (n = 70 ROIs from 51 neurons), respectively. In addition, we created maximum projections from the Z-stacks to eliminate any potential bias due to epifluorescent focusing. We found similar results, with a mean control G/F-actin ratio of 0.834, whereas 5-HT and DPAT treatment increased ratios to 2.56 and 2.823, respectively.

In these experiments, higher ratios correlate with either an increase in G-actin and/or a decrease in F-actin. The interpretation of these higher ratios, therefore, is that 5-HT and DPAT induced actin depolymerization. Previous studies have shown that 5-HT1A activation in serotonergic neurons can modulate ERK1/2 via several mechanisms, including b-arrestin scaffolding (Della Rocca et al., 1999), heterodimerization with FGFRs (Borroto-Escuela et al., 2013, 2015), and alternative G-protein subunit coupling (Albert & Vahid-Ansari, 2019; Kushwaha & Albert, 2005; Rojas & Fiedler, 2016). ERK1/2 signaling has been shown to regulate actin dynamics through phosphorylation of RhoA (Kim et al., 2004; Kumar et al., 2005), highlighting its potential role in 5-HT1A mediated F-actin depolymerization. The increase in G/F-actin ratio observed upon addition of 5-HT or DPAT supports a model in which 5-HT1A signaling induces F-actin depolymerization, possibly through downstream ERK1/2 activation and subsequent modulation of actin regulator proteins such as Rho GTPases. Consistent with our previous work, we found the distribution of our negative control data to be normal, whereas treatment of 5-HT and DPAT causes a shift to a non-normal distribution. We speculate here that this occurs because only a subset of serotonergic neurons expresses 5-HT1A in this system, and therefore only a subpopulation of axons responds. However, this should be tested in future experiments using 5-HT1A expression markers to delineate these subpopulations.

## Methods

Primary neuronal culture of serotonergic neurons was performed as described previously (Long et al., 2023). In brief, stage 11 *Drosophila* embryos were collected and dechorionated in 50% bleach before cells were aspirated and transferred to Schnieder’s *Drosophila* medium (Fisher 21-720-024) supplemented with 20% FBS (Fisher 16-140-063), 0.02% P/S, and 0.2 mg/mL insulin (Sigma 10515-5ML). The cells were chemically digested with collagenase followed by physical trituration before plating on 0.01% poly-L-lysine treated coverslips. Serotonin hydrochloride (Sigma H9523) was suspended at 10mM in sterile PBS prior to suspending in media at 100 μM final concentration. The 5-HT1A agonist 8-hydroxy-DPAT hydrobromide (Tocris Bioscience 0529) was suspended in DMSO prior to diluting to final concentration of 10&nbsp;μM in 1&nbsp;mL of culture media. This resulted in a final percentage of 0.01 DMSO in culture media. Drugs were added to cultures 2 hours prior to fixing. Cells were fixed with 4% paraformaldehyde for 15 min at room temperature, washed three times with PBS for 3 min, then incubated with rabbit anti-serotonin (ImmunoStar 20080) at 1:500 dilution in 1× PBS with 0.1% Triton X-100 for 45&nbsp;min at room temperature. After washing for 5&nbsp;min in 1× PBS with 0.1% Triton X-100 three times, samples were incubated in donkey anti-rabbit IgG AMCA (Jackson ImmunoResearch 711-155-152) at 1:500 dilution, 0.4 mg/mL SiR-Actin, (Cytoskeleton CYSC001), and 0.4 mg/mL DNAse I-Alexa488 (Invitrogen D12371) in 1× PBS for 30&nbsp;min at room temperature. Neurons were washed three times in 1× PBS for 10&nbsp;min at room temperature before being briefly rinsed in Milli-Q water. Samples were mounted in Vectashield Antifade Mounting Medium (Vector Labs H-1000).

Neurons were imaged on a Zeiss Apotome Axio Imager.M2 with a 63x NA 1.4 objective, X-cite 120LED Lamp, and Zeiss 807 mono camera. AMCA was excited at 353 nm with emission collected at 465 nm. SiR-actin was excited at 650 nm with emission collected at 673nm. DNAse I was excited at 488 nm with emission collected at 509 nm. Serotonergic neurons were identified using fluorescent AMCA channel. In FIJI, the background was subtracted from a composite image of all three channels, with the best focal plane for the actin channels selected for analysis. For each neuron, the distal 5 μm tip of each axon branch was selected as an ROI and used to measure the mean pixel intensity for each actin channel in the selected area. The G/F actin ratio at each distal axon tip was determined by dividing the mean pixel intensity of G-actin by the mean pixel intensity of F-actin (Torreano et al., 2005). In addition, maximum projections of the optical sectioning stacks were created and analyzed as above. All statistical analyses were conducted in GraphPad Prism. Normality was tested using the Shapiro–Wilk test. An analysis of variance was run on the G/F-actin ratios with Kruskal-Walis post hoc comparisons.
